# Inferring Pareto-optimal reconciliations across multiple event costs under the duplication-loss-coalescence model

**DOI:** 10.1186/s12859-019-3206-6

**Published:** 2019-12-17

**Authors:** Ross Mawhorter, Nuo Liu, Ran Libeskind-Hadas, Yi-Chieh Wu

**Affiliations:** Department of Computer Science, Harvey Mudd College, Claremont, 91711 CA USA

**Keywords:** Phylogenetics, Reconciliation, Coalescence, Incomplete lineage sorting, Gene duplication and loss, Pareto optimality

## Abstract

**Background:**

Reconciliation methods are widely used to explain incongruence between a gene tree and species tree. However, the common approach of inferring maximum parsimony reconciliations (MPRs) relies on user-defined costs for each type of event, which can be difficult to estimate. Prior work has explored the relationship between event costs and maximum parsimony reconciliations in the duplication-loss and duplication-transfer-loss models, but no studies have addressed this relationship in the more complicated duplication-loss-coalescence model.

**Results:**

We provide a fixed-parameter tractable algorithm for computing Pareto-optimal reconciliations and recording all events that arise in those reconciliations, along with their frequencies. We apply this method to a case study of 16 fungi to systematically characterize the complexity of MPR space across event costs and identify events supported across this space.

**Conclusion:**

This work provides a new framework for studying the relationship between event costs and reconciliations that incorporates both macro-evolutionary events and population effects and is thus broadly applicable across eukaryotic species.

## Background

Phylogenetic tree reconciliation is a fundamental technique for studying the evolution of gene families. Given a gene tree, a species tree, and an association between their leaves, reconciliation methods explain the incongruence between the trees by postulating a sequence of evolutionary events, with different evolutionary models allowing for different types of events. For example, the duplication-loss (DL) model [1, 2] allows for gene duplication and gene loss, the duplication-transfer-loss (DTL) model [3, 4] allows for horizontal gene transfers as well, and the multispecies coalescent (MSC) model [5] allows for incomplete lineage sorting through deep coalescence. However, the DL and DTL models do not model population effects, and the MSC model implicitly assumes that all genes are orthologs.

More recently, several combined duplication-loss-coalescence (DLC) models have been developed, which, as the name implies, allow for duplication, loss, and coalescence. Little evidence has been found for horizontal gene transfer in eukaryotes [6], making DLC-models suitable for capturing eukaryotic evolution. In this work, we rely on the DLCoal model of Rasmussen and Kellis [7]. While the models of Vernot et al. [8] and Chan et al. [9] are conceptually simpler, neither keep track of the inferred loci of genes nor rely explicitly on the multispecies coalescent, limitations that prevent the models from capturing all possible evolutionary histories [10, 11]. (For a detailed comparison of these models, see Chan et al. [9] and Du et al. [11].) While it is possible to perform DLC reconciliation using a probabilistic approach [7], for efficiency and broad applicability, we use a maximum parsimony framework, in which each type of event in the model has an associated user-defined cost and the objective is to find a reconciliation of minimum total cost. In prior work, we introduced a new structure for representing reconciliations and an algorithm DLCpar for inferring a maximum parsimony reconciliation (MPR) [10].

However, while it is generally understood that MPRs are sensitive to event costs, analyses typically choose a single setting of costs for each type of event. Probabilistic approaches can weight events by estimating event rates and population parameters [7], but there is currently no systematic method for choosing event costs or determining the relationship between event costs and the resulting MPRs under the DLC model.

The problem of appropriate event costs does not arise in the DL model, as the MPR is always unique if duplication and loss events have positive costs [12]. For the DTL model, several authors use Pareto-optimality, modifying existing dynamic programming algorithms for DTL reconciliation to compute event counts rather than reconciliation costs [3, 13–15]. We build on their ideas to apply the same concept to the considerably more complicated DLCpar algorithm. In addition, we demonstrate how to track events across Pareto-optimal solutions; such an extension was mentioned but not detailed in our prior work on the DTL model [14]. Tracking events substantially complicates the algorithm; we formally elaborate on this process for the DLC model.

In summary, the contributions of this paper are as follows:

- We provide an algorithm DLCparETO that extends DLCpar to compute Pareto-optimal event counts over a range of event costs. Our algorithm also counts the number of distinct reconciliations associated with each event count and records all events that arise in those reconciliations, along with their frequencies.
- We demonstrate how DLCparETO can be used to partition the space of event cost parameter values into regions such that all sets of event costs in a given region result in the same set of maximum parsimony reconciliations. In addition, we demonstrate how to compute support for individual events.
- We analyze our algorithms and show that they are efficient except when the two trees are extremely incongruent.

We have applied our algorithms to a biological dataset of 16 fungal species [16] to gain insight into the effect of event costs on maximum parsimony reconciliations and to compute several measures of support for constituent events.

## Methods

### Preliminaries

We start by reviewing prior work that formalizes the concept of reconciliations and maximum parsimony reconciliations under the DLC model [10, 11]. For brevity, we provide an overview of the key concepts here; formal definitions appear in Additional file 1: Section S1.

**Fig. 1:**
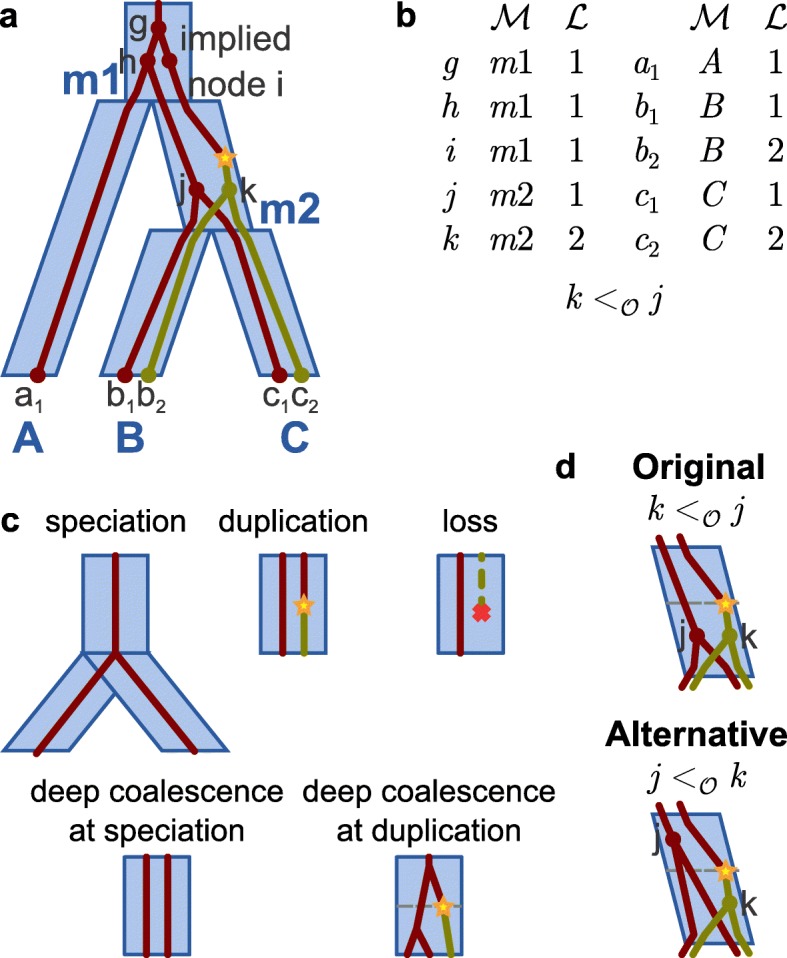
The labeled coalescent tree. **a** Evolution is represented using the LCT. In this example, a duplication (yellow star) creates a new locus, “locus 2” (yellow), from the original locus, “locus 1” (red), and lineages *j* and *k* fail to coalesce within species *m*2. **b** The LCT consists of a species map $\mathcal {M}$, a locus map $\mathcal {L}$, and a partial order $\mathcal {O}$. **c** Evolutionary events are depicted in the LCT. Except for speciation, evolution within a single species tree branch is shown. **d** An alternative scenario is presented for evolution in species *m*2. The new partial order induces an extra lineage at the time of the duplication. [Figure and caption adapted with permission from Du et al. [11] and Wu et al. [10]]

Given a gene tree *G*, a species tree *S*, and a leaf mapping *Le* from the leaves of *G* to the leaves of *S* (which need not be one-to-one nor onto), a reconciliation seeks to map *G* “inside” *S*. The *labeled coalescent tree* (LCT, Fig. 1a, Additional file 1: Section S1.3) formalizes this notion of a reconciliation in the DLC model.

Given *G*, *S*, and *Le*, an LCT for 〈*G*,*S*,*Le*〉 is a tuple $\langle \mathcal {M}, \mathcal {L}, \mathcal {O} \rangle $, where $\mathcal {M}$ is a *species map* that maps each node of *G* to a node of *S*; $\mathcal {L}$ is a *locus map* that maps each node of *G* to a finite set of natural numbers, each representing a locus that has evolved within the gene family; and $\mathcal {O}$ is a *partial order* that orders gene tree nodes within the same species and locus (Fig. 1b). Note that the mapping $\mathcal {M}$ is defined first, then implied nodes are added to *G* so that each gene branch spans only a single branch of the species tree, then $\mathcal {L}$ is defined, and finally $\mathcal {O}$ is defined.

It will be convenient to consider the restriction of an LCT to a single species branch or to a subtree rooted at a species, in each case considering only the parts of the gene tree that evolve within the considered species. Henceforth, the term LCT encompasses these restrictions.

Given a species node *s* and a species map $\mathcal {M}$, let *nodes*(*s*) denote the set of gene nodes that map to *s*; *bottoms*(*s*) denote the subset of *nodes*(*s*) that are leaves or whose children map to descendants of *s*; and *tops*(*s*)=*bottoms*(*p*(*s*)) if *s*≠*r*(*S*) and *tops*(*s*)={*r*(*G*)} otherwise, where *p*(*s*) denotes the parent of *s* and *r*(*S*) and *r*(*G*) denote the roots of the species tree and gene tree, respectively. Note that *bottoms*(*s*) and *tops*(*s*) can be viewed as the set of gene nodes at the “bottom” or “top” of the branch for species *s*, respectively.

The LCT allows for several evolutionary events (Fig. 1c, Additional file 1: Section S1.4). A *speciation* event corresponds to a locus present at the bottom of a species branch continuing at the same locus in at least one child species. As a speciation in the LCT reflects a speciation in the species tree, it is considered a null event. A *duplication* event corresponds to the creation of a new locus along a gene branch, which occurs when a gene node and its parent are mapped to different loci; such a gene branch is said to have a duplication. A *loss* event corresponds to a locus present at either the top of a species branch, or created via a duplication within the species branch, being no longer present at the bottom of the species branch. A *coalescence* event is, in fact, a *deep coalescence*, in which two or more lineages fail to coalesce; such failure can result in multiple lineages at speciations or duplications. Note that counting speciation, duplication, loss, and coalescence at speciation events requires only the species map and locus map while counting coalescence at duplication events also requires the partial order (Fig. 1d).

Let *C*_*D*_,*C*_*L*_, and *C*_*C*_ denote the positive real-number costs associated with duplication, loss, and coalescence events, respectively. (Separate costs can be associated with the two types of coalescence events, as well.) The cost of reconciling *G* and *S* according to LCT $\langle \mathcal {M},\mathcal {L},\mathcal {O} \rangle $ is defined as follows:

#### **Definition 1**

Reconciliation Cost Given *G*, *S*, *Le*, *C*_*D*_,*C*_*L*_, and *C*_*C*_, the *reconciliation cost* of an LCT $\langle \mathcal {M},\mathcal {L},\mathcal {O} \rangle $ for 〈*G*,*S*,*Le*〉 with *d* duplication events, *ℓ* loss events, and *c* coalescence events is $\mathcal {R}_{\langle \mathcal {M},\mathcal {L},\mathcal {O} \rangle } = d \cdot C_{D}+ \ell \cdot C_{L} + c \cdot C_{C}$.

Given *G*, *S*, *Le*, *C*_*D*_,*C*_*L*_, and *C*_*C*_, the objective of the most parsimonious reconciliation (MPR) problem is to find an LCT for 〈*G*,*S*,*Le*〉 with minimum reconciliation cost (Additional file 1: Section S1.5). The solution to this problem is not necessarily unique.

Next, we define optimality of LCT components.

#### **Definition 2**

(Optimal LCT Components) A species map $\mathcal {M}^{*}$ is said to be optimal if there exists a locus map $\mathcal {L}$ and a partial order $\mathcal {O}$ such that $\langle \mathcal {M}^{*},\mathcal {L},\mathcal {O}\rangle $ solves the MPR problem. Given a species map $\mathcal {M}$, a locus map $\mathcal {L}^{*}$ is said to be optimal if there exists a partial order $\mathcal {O}$ such that $\langle \mathcal {M},\mathcal {L}^{*},\mathcal {O}\rangle $ solves the MPR problem. Given a species map $\mathcal {M}$ and locus map $\mathcal {L}$, a partial order $\mathcal {O}^{*}$ is said to be optimal if $\langle \mathcal {M},\mathcal {L},\mathcal {O}^{*}\rangle $ solves the MPR problem.

Henceforth, the term MPR refers to an LCT that solves the MPR problem. We previously showed that the species map $\mathcal {M}^{*}$ is optimal if and only if $\mathcal {M}^{*}$ is the lowest common ancestor (LCA) map [10, 11].

### Problem statement and definitions

The MPR problem requires that event costs be specified. As appropriate event costs can be difficult to estimate, we seek to solve the MPR problem when event costs are not known *a priori*.

Let [*d*_min_,*d*_max_],[*ℓ*_min_,*ℓ*_max_], and [*c*_min_,*c*_max_] be ranges of positive real-number costs associated with duplication, loss, and coalescence events, respectively. We seek to solve the equivalent region partition problem.

#### **Problem 1**

(Equivalent Region Partition (ERP)) Given *G*, *S*, *Le*, [*d*_min_,*d*_max_],[*ℓ*_min_,*ℓ*_max_], and [*c*_min_,*c*_max_], partition the space of event costs [*d*_min_,*d*_max_]×[*ℓ*_min_,*ℓ*_max_]×[*c*_min_,*c*_max_] into a finite number of equivalence classes, or *regions*, such that all event costs within the same region yield the same set of MPRs.

Note that the regions are not strictly speaking partitions of the space since regions may overlap at boundaries, and thus a given point in event cost space may be an element of multiple regions.

We also wish to identify events that are highly supported by merit of occurring in a large fraction of MPRs or a large fraction of regions. We therefore collect the set of events that are in *any* MPR in a region.

Our algorithm for solving the ERP problem and computing event support requires new data types and operations. In particular, rather than optimizing for reconciliation cost, we now focus on inferred event counts and Pareto-optimality.

#### **Definition 3**

(Event Count) Given *G*, *S*, and *Le*, the *event count* of an LCT $\langle \mathcal {M}, \mathcal {L}, \mathcal {O} \rangle $ for 〈*G*,*S*,*Le*〉 with *d* duplication events, *ℓ* loss events, and *c* coalescence events is the vector 〈*d*,*ℓ*,*c*〉.

Given two event counts *v*=〈*d*,*ℓ*,*c*〉 and *v*^′^=〈*d*^′^,*ℓ*^′^,*c*^′^〉, *v* is said to be *strictly better* than *v*^′^ if each entry of *v* is less than or equal to the corresponding entry in *v*^′^ and at least one entry of *v* is less than its corresponding entry in *v*^′^.

Given a set *A* of event counts, an event count *v*∈*A* is said to be *Pareto-optimal* with respect to *A* if there does not exist any other *v*^′^∈*A* that is strictly better than *v*. The set *A* is said to be *Pareto-optimal* if every event count in *A* is Pareto-optimal with respect to *A*. Note that an LCT with a non-Pareto-optimal event count cannot be an MPR under any event costs.

Given a set of LCTs, multiple LCTs in the set may yield the same event count. Therefore, we define a structure to keep track of the number of LCTs with the same event count. To compute event support, we also keep track of the specific events and frequencies.

#### **Definition 4**

(Event Count Descriptor) Given *G*, *S*, and *Le*, an *event count descriptor* for 〈*G*,*S*,*Le*〉 is a tuple 〈*v*,*κ*,*E*〉, where

- *v*=〈*d*,*ℓ*,*c*〉 is an **event count**.
- *κ* is the number of LCTs with event count *v*, called the **LCT count**.
- *e* is a set, called the **event set**, of ordered pairs of the form (*e*,*k*), where *e* is an event that occurs in some LCT with event count *v* and *k* is the number of those LCTs that contain that event.

For simplicity, we will often use the short-hand *descriptor* to refer to an event count descriptor.

Given a set $\mathcal {A}$ of descriptors, let $v(\mathcal {A}), \kappa (\mathcal {A})$, and $E(\mathcal {A})$ denote the sets of event counts, LCT counts, and event sets in $\mathcal A$. A descriptor $w \in \mathcal {A}$ is said to be *Pareto-optimal* with respect to $\mathcal {A}$ if the event count of *w* is Pareto-optimal with respect to $v(\mathcal {A})$. The set $\mathcal {A}$ is said to be *Pareto-optimal* if every descriptor in $\mathcal {A}$ is Pareto-optimal with respect to $\mathcal {A}$. Given a set $\mathcal A$ of descriptors, the term *Pareto-optimal subset of* $\mathcal A$ is the unique set that results from removing all descriptors that are not Pareto-optimal with respect to $\mathcal {A}$.

Next, we define operations on these new data types. These operations will be used when merging subproblems in our extension to DLCpar. Recall that an LCT may refer to the restriction to a species branch or to a subtree rooted at a species. Let $\mathcal {A}$ and $\mathcal {B}$ be two sets of Pareto-optimal descriptors for sets *A* and *B* of LCTs. Then, let $\mathcal {A} \oplus \mathcal {B}$ be the Pareto-optimal subset of $\mathcal {A} \cup \mathcal {B}$. This subset describes the union *A*∪*B* of LCTs, with the restriction that an LCT in this union have a Pareto-optimal event count. Similarly, let $\mathcal {A} \otimes \mathcal {B}$ be the Pareto-optimal subset of $\mathcal {A} \times \mathcal {B}$, where × indicates the Cartesian product. This subset describes the Cartesian product *A*×*B* of LCTs that combine one LCT from *A* and one LCT from *B*, with the restriction that the resulting LCTs have a Pareto-optimal event count. In the interest of precision, we now provide formal definitions of these two operations.

To start, we define operations on event counts and event sets. Given two event counts *v*=〈*d*,*ℓ*,*c*〉 and *v*^′^=〈*d*^′^,*ℓ*^′^,*c*^′^〉, let

$$v + v' = \langle d + d', \ell + \ell', c+ c' \rangle. $$

Given an event set *E* and a positive integer *x*, let

$$x \cdot E = \{ (e, kx) \mid (e, k) \in E\}; $$

that is, the count *k* of each event *e* is increased by a factor of *x*. Given two event sets *E* and *F*, let *E*⊕*F* be the union of the sets; that is, the union of the two sets of events, with the corresponding counts added together. Let

$$P = \{ (e, x + y) \mid (e,x) \in E ;\ (e,y) \in F \} $$

contain the set of events that appear in both sets, with their counts from both sets added, and let

$$Q = \{(e, x) \mid (e,x) \in E \cup F ;\ \nexists y \textrm{ s.t.} (e,y) \in P \} $$

contain the set of events that appear in only one set, with their original counts. Then *E*⊕*F*=*P*∪*Q*.

Next, we define operations on descriptors. Recall that $\mathcal {A} \oplus \mathcal {B}$ is the Pareto-optimal subset of $\mathcal {A} \cup \mathcal {B}$ and describes the union *A*∪*B* of LCTs, such that an LCT in this set has a Pareto-optimal event count. Thus, $\mathcal {A} \oplus \mathcal {B}$ is the set obtained by taking the union of the component sets, then removing the elements that are not Pareto-optimal with respect to that set. Similar to our operation of ⊕ for event sets, let

$$\mathcal{P} = \{ \langle v, \kappa + \kappa', E \oplus E' \rangle \mid \langle v, \kappa, E \rangle \in \mathcal{A} ;\ \langle v, \kappa', E' \rangle \in \mathcal{B} \} $$

combine descriptors whose event counts appear in both sets, and let

$$\mathcal{Q} = \{ \langle v, \kappa, E \rangle \mid \langle v, \kappa, E \rangle \in \mathcal{A} \cup \mathcal{B} ;\ v \notin v(\mathcal{P}) \} $$

contain descriptors whose event counts appear in only one set. Then $\mathcal {A} \oplus \mathcal {B}$ is the Pareto-optimal subset of $\mathcal {P} \cup \mathcal {Q}$.

Similarly, recall that $\mathcal {A} \otimes \mathcal {B}$ is the Pareto-optimal subset of $\mathcal {A} \times \mathcal {B}$ and describes the Cartesian product *A*×*B* of LCTs, such that an LCT in this set has a Pareto-optimal event count. Thus, $\mathcal {A} \otimes \mathcal {B}$ is the set obtained by first computing the Cartesian product of the component sets, then converting each resulting ordered pair into a single descriptor, and finally removing the elements that are not Pareto-optimal with respect to that set. Given two descriptors *a*=〈*v*,*κ*,*E*〉 and *b*=〈*v*^′^,*κ*^′^,*E*^′^〉, let

$$a + b = \langle v + v', \kappa \cdot \kappa', \kappa' \cdot E \oplus \kappa \cdot E' \rangle $$

be the combined descriptor. The event counts add because the combined LCT includes events from both component LCTs, and the LCT counts multiply from combining one of *κ* LCTs with one of another *κ*^′^ LCTs. Finally, each LCT with event *e* in event set *E* is combined with one of *κ*^′^ LCTs; hence the count for *e* is increased by a factor of *κ*^′^ (and similarly for event *e*^′^ in event set *E*^′^). Next, let

$$\begin{array}{*{20}l} \mathcal{R} = \{a + b \mid & a \in \mathcal{A} ;\ b \in \mathcal{B} \} \end{array} $$

combine all descriptors from $\mathcal {A}$ and $\mathcal {B}$. Lastly, we must merge descriptors that share event counts. For $v \in v(\mathcal {R})$, let $\mathcal {T}(v)$ denote the subset of descriptors $\langle v', \kappa ', E' \rangle \in \mathcal {R}$ such that *v*^′^=*v*. Then $\mathcal {A} \otimes \mathcal {B}$ is the Pareto-optimal subset of

$$\mathcal{S} = \{ \langle v, \textstyle \sum {\kappa(\mathcal{T}(v))}, \oplus {E(\mathcal{T}(v))} \rangle \mid v \in v(\mathcal{R}) \}. $$

Note that the computation of set $\mathcal {S}$ is a generalization of $\mathcal {P} \cup \mathcal {Q}$ for $\mathcal {A} \oplus \mathcal {B}$.

### DLCparETO algorithm

**Fig. 2:**
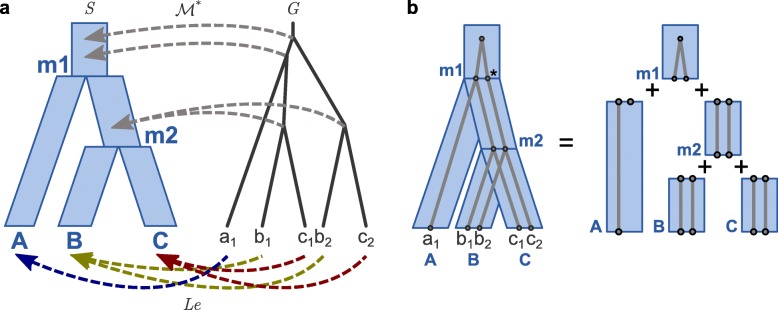
The DLCparETO algorithm, decomposing the gene tree. **a** The input species tree *S*, gene tree *G*, and leaf mapping *Le*. The optimal species map $\mathcal {M}^{*}$ is the LCA map. **b** The gene tree decomposed by species. Implied nodes (∗) are added to gene tree branches that span multiple branches of the species tree. [Figure and caption adapted with permission from Du et al. [11] and Wu et al. [10]]

**Fig. 3:**
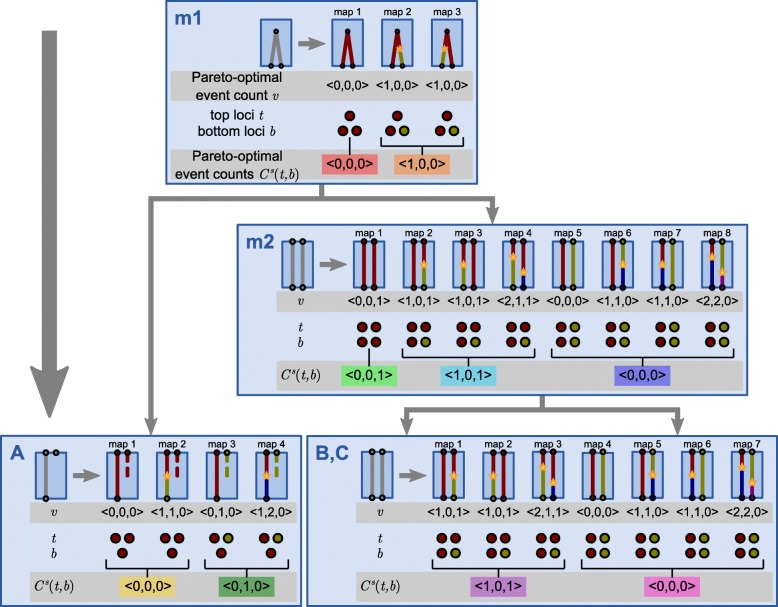
The DLCparETO algorithm, enumerating tiles. Example continued from Fig. 2. Tiles for each species enumerated via pre-order traversal of the species tree. Each tile consists of a sub-locus map with a singleton set of one event count *v*=〈*d*,*ℓ*,*c*〉 comprising the number of duplications, losses, and coalescences. *v* is Pareto-optimal for the sub-locus map across all partial orders. (The algorithm tracks descriptors rather than event counts, but for simplicity, only event counts are shown.) To propagate locus assignments across species, the set of top loci *t* and bottom loci *b* for each tile are compactly represented. Each unique relative locus pair (*t*,*b*) is associated with a set *C*^*s*^(*t*,*b*) of Pareto-optimal event counts. In this example, species *B* and *C* have the same set of tiles, though in practice, these tiles are enumerated separately. [Figure and caption adapted with permission from Du et al. [11] and Wu et al. [10]. Gray boxes indicate new content]

**Fig. 4:**
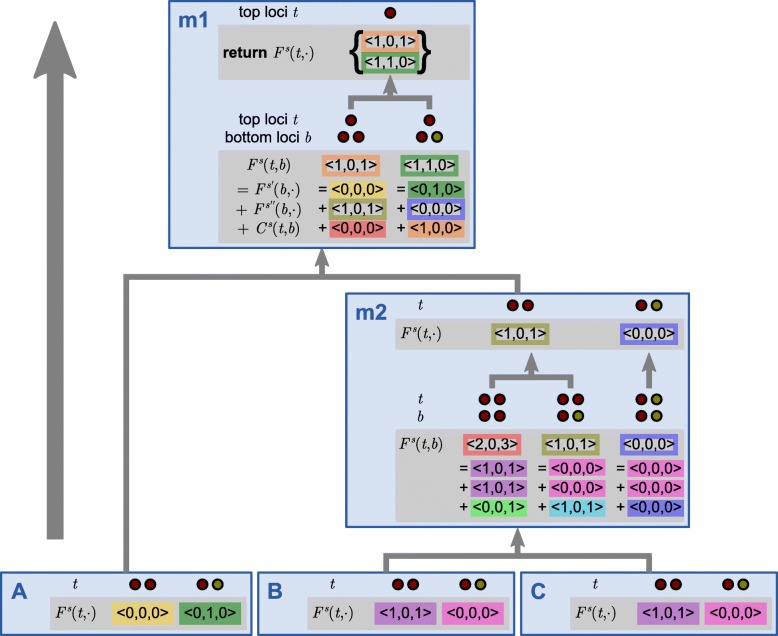
The DLCparETO algorithm, stitching together tiles. Example continued from Figs. 2 and 3. The dynamic programming structure for merging Pareto-optimal event counts across species, completed via post-order traversal of the species tree. Solutions *F*^*s*^(*t*,·) (and *F*^*s*^(*t*,*b*)) for sub-problems denote the set of Pareto-optimal event counts for the subtree rooted at species *s* with top loci *t* (and bottom loci *b*). Colored boxes indicate which tile from Fig. 3 and which bottom loci are used. The algorithm terminates at the species tree root and returns the set of Pareto-optimal event counts for the reconciliation of *G* and *S*. [Figure and caption adapted with permission from Du et al. [11] and Wu et al. [10]. Gray boxes indicate new content]

**Fig. 5:**
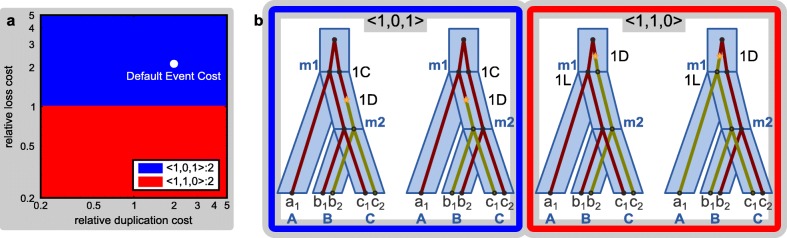
Equivalent regions and maximum parsimony reconciliations. Example continued from Figs. 2, 3, and 4. **a** The landscape of equivalent regions. Regions are polygons, lines, or points; colors are arbitrary and are used to match regions with the event counts in the legend. In addition, the number of LCTs with each event count, and the default cost setting for DLCpar (*C*_*D*_=1,*C*_*L*_=1,*C*_*C*_=0.5) are shown. **b** Most parsimonious reconciliations for each region. DLCparETO does not return these MPRs, but for a fixed setting of event costs, DLCpar returns an MPR sampled uniformly at random. [Figure and caption adapted with permission from Du et al. [11] and Wu et al. [10]. Gray boxes indicate new content]

We now describe the basic steps of the DLCparETO algorithm for tracking Pareto-optimal descriptors (Figs. 2, 3, 4, and 5). Formal pseudocode is provided in Additional file 1: Section S2. Note that Pareto-optimality is defined independently of event cost ranges. We will later show how event cost ranges are used together with this algorithm to solve the ERP problem. DLCparETO is based on an extension of DLCpar [10, 11], but rather than returning a single optimal LCT, the goal of DLCparETO is to return a set of Pareto-optimal descriptors that correspond to one more LCTs, each of which is optimal for some setting(s) of event costs.

Given *G*, *S*, and *Le*, DLCparETO sets the optimal species map $\mathcal {M}^{*}$ to be the LCA map (Fig. 2a), then prunes the species tree to the subtree rooted at $\mathcal {M}^{*}(r(G))$. Next, the algorithm uses this map to decompose the gene tree into disjoint forests that evolve within each species branch (Fig. 2b). For each species node *s*, let a *sub-locus map* and *sub-partial order* be a locus map and partial order restricted to the gene nodes in the species branch, that is, over *g*∈*tops*(*s*)∪*nodes*(*s*). Let a *tile* consist of a particular sub-locus map with its associated descriptor. DLCparETO constructs a set of tiles for each species, then uses dynamic programming to “stitch” together tiles across species such that loci of nodes shared across species match. Each stitch combines tiles and thus must also merge the corresponding sets of descriptors. In the remainder of this section, we provide more details on this process.

DLCparETO traverses the species tree in pre-order and for each valid sub-locus map, computes a set $\mathcal {A}$ of Pareto-optimal descriptors as follows (Fig. 3). $\mathcal {A}$ is initially empty. To enumerate sub-locus maps, consider as an example the root species, which contains a part of the gene tree. DLCparETO assigns the root of the tree to an arbitrary locus, then considers all possible placements of duplications along branches, subject to the constraints on an LCT, with each combination of duplication placements yielding a sub-locus map. For each sub-locus map, DLCparETO considers all valid sub-partial orders. For each sub-locus map and sub-partial order, it then computes the set of induced events and constructs a descriptor *a* comprising the event count, an LCT count of 1, and an event set comprising pairs (*e*,1) for each event. The algorithm then updates $\mathcal {A}$ to $\mathcal {A} \oplus \{a\}$. Note that because a sub-partial order affects only the number of coalescence at duplication events, each sub-locus map induces the same number of duplication and loss events and therefore has a single Pareto-optimal event count and descriptor. Thus, after this update, $\mathcal {A}$ is always a singleton set. If a species branch contains no gene tree nodes, then $\mathcal {A}$ contains a single descriptor with an event count of 〈0,0,0〉, an LCT count of 1, and an empty event set.

Next, DLCparETO considers the problem of propagating locus assignments across species. For each sub-locus map, the algorithm computes *top loci* and *bottom loci*, which are compact representations of the locus assignments at *tops*(*s*) and *bottoms*(*s*). As in DLCpar, the algorithm constructs these representations by arbitrarily (but consistently) ordering *tops*(*s*) (or *bottoms*(*s*)), assigning the first node to an arbirary “locus 1”, then assigning each subsequent node either to one of the previous loci, if the node is mapped to the same locus as a previous node, or to the next available locus. The *relative locus pair* for a sub-locus map is a tuple (*t*,*b*) with top loci *t* and bottom loci *b*, each of which are a sequence of relative locus numbers. Let *C*^*s*^(*t*,*b*) denote the set of Pareto-optimal descriptors for a tile for species *s* with relative locus pair (*t*,*b*). DLCparETO constructs these sets as follows. *C*^*s*^(*t*,*b*) is initially empty. For each tile with a set $\mathcal {A}$ of descriptors, the algorithm determines the relative locus pair (*t*,*b*) induced by the tile, then updates *C*^*s*^(*t*,*b*) to $C^{s}(t,b) \oplus \mathcal {A}$. Note that by traversing the species tree in pre-order, DLCparETO ensures that the set of top loci for any non-root species is determined by the set of bottom loci of its parent species, and the set of bottom loci for any species is in turn determined by the sets of top loci and enumerated sub-locus maps for the species.

Once all tiles are constructed for all species, DLCparETO uses dynamic programming to merge sets of descriptors across species as follows (Fig. 4). Let **RLP**(*s*) denote the set of relative locus pairs for species *s*, and let *F*^*s*^(*t*,·) denote the set of Pareto-optimal descriptors for the subtree rooted at species *s* with top loci *t* for *s*. (Note that this latter set includes evolution within species *s* but not evolution within the sibling of *s*.) DLCparETO traverses the species tree in post-order and for each species *s*, considers the possible top loci {*t*∣(*t*,*b*)∈**RLP**(*s*)} for the species. If *s* is a leaf, the subtree rooted at *s* is simply the node *s*. Furthermore, DLCparETO has already required that bottom loci for extant species be distinct when enumerating valid sub-locus maps, so there exists only one possible assignment *b* of bottom loci. Therefore,

$$F^{s}(t,\cdot) = C^{s}(t,b). $$

If *s* is not a leaf, then *F*^*s*^(*t*,·) relies on a helper variable *F*^*s*^(*t*,*b*) that denotes the set of Pareto-optimal descriptors for the subtree rooted at *s* with top loci *t* and bottom loci *b* for *s*. Note that *F*^*s*^(*t*,*b*) requires assigning top loci *b* to children species *s*^′^ and *s*^′′^ and a tile for *s* with relative locus pair (*t*,*b*). Therefore,

$$F^{s}(t,b) = F^{s'}(b,\cdot) \otimes F^{s^{\prime\prime}}(b,\cdot) \otimes C^{s}(t,b). $$

Then *F*^*s*^(*t*,·) must choose among bottom loci for the species,

$$F^{s}(t,\cdot) = \oplus_{b : (t,b) \in \mathbf{RLP}(s)}{F^{s}(t,b)}. $$

Once the species tree root *s*=*r*(*S*) is reached, there is only one possible assignment *t* of top loci, so DLCparETO returns *F*^*s*^(*t*,·).

### Computing regions

From Libeskind-Hadas et al. [14], the set of Pareto-optimal event counts from DLCparETO can be used to solve the ERP problem, that is, to partition the space of event costs into equivalent regions (Fig. 5a). For completeness, this algorithm is described below. To allow visualization in two dimensions, we rely on the costs being unit-less to normalize the coalescence cost to 1 and consider the costs of duplication and loss to be positive values relative to the unit cost of coalescence. An event count *v*=〈*d*,*ℓ*,*c*〉 with positive real-number costs *C*_*D*_ and *C*_*L*_ for duplication and loss events, respectively, has a reconciliation cost $\mathcal {C}(v,C_{D},C_{L}) = d \cdot C_{D} + \ell \cdot C_{L} + C_{C}$. A set *A* of Pareto-optimal event counts induces a partition of the event cost space into regions, where region *R*(*v*) associated with event count *v*∈*A* is the set of points (*C*_*D*_,*C*_*L*_)∈[*d*_min_,*d*_max_]×[*ℓ*_min_,*ℓ*_max_] such that $\mathcal {C}(v, C_{D}, C_{L}) \leq \mathcal {C}(v', C_{D}, C_{L}) \ \forall v' \in A - v$. Because each inequality induces a half-space, a region is the intersection of several half-spaces. Note that not all Pareto-optimal event counts induce a minimum reconciliation cost for a given set of event costs [15]. In this work, we consider only the event counts that are Pareto-optimal for the given space of event costs.

### Time complexity

Let *m* denote the number of leaves in the species tree, *n* denote the number of leaves in the gene tree, and *k* denote the maximum number of nodes at the top or bottom of any species branch. In this section, we show that the ERP problem is fixed-parameter tractable by showing that the running time of DLCparETO is **O**(*f*(*k*)*m*^7^) for some function *f* that depends only on *k*.

Note that the value of *k* is not inherent to the gene tree or species tree but rather is induced by the LCA species map. When the gene and species trees are congruent, *k*=1, and in general, when the trees are not highly discordant, *k* is small. However, in the worst case, *k*=*n*, and since the function *f*(*k*) has fast asymptotic growth in *k*, this algorithm may not be viable for large and highly discordant trees.

Although it is possible to derive an explicit closed-form for the function *f*(*k*), it is not necessary for establishing fixed-parameter tractability and is therefore omitted in the interest of brevity. For the simpler DLCpar algorithm, *f*(*k*)=*B*_*k*_2^2*k*^(2*k*)!*k*^2^, where *B*_*k*_ denotes the *k*^*th*^ Bell number [11]. The function *f*(*k*) for DLCparETO involves similar terms but is considerably more complicated.

By assumption, the parts of the gene tree that exist within each species branch form a forest with at most *k* roots and *k* leaves; thus, the forest contains **O**(*k*) gene tree nodes and branches. We will use this observation repeatedly in the proofs of the results below.

#### **Lemma 1**

The cardinality of any set of Pareto-optimal descriptors is bounded by **O**(*g*(*k*)*m*^2^) for some function *g*(*k*).

#### *Proof*

The number of Pareto-optimal descriptors is the number of Pareto-optimal event counts. Consider a single species branch which, as noted above, has **O**(*k*) gene tree nodes and branches. Each gene branch can have a duplication for a bound of **O**(*k*) duplications per species. Since there are at most **O**(*k*) gene nodes, and each node can map to a different loci, the number of losses per species is also bounded by **O**(*k*). Therefore, across all *m* species, there are at most **O**(*km*) duplications and **O**(*km*) losses. A Pareto-optimal set of event counts may contain at most one event count vector 〈*d*,*ℓ*,*c*〉 for a given pair *d*,*ℓ*. Thus, the number of Pareto-optimal descriptors is bounded by **O**((*km*)^2^) which is **O**(*g*(*k*)*m*^2^) for *g*(*k*)=*k*^2^. □

#### **Lemma 2**

The number of events in the event set of a Pareto-optimal descriptor is bounded by **O**(*h*(*k*)*m*) for some function *h*(*k*).

#### *Proof*

In an optimal LCT, the species map is fixed. Consider a single species branch which, as noted above, has **O**(*k*) gene tree nodes. Since the events are induced solely by the loci and orderings of nodes (in addition to the fixed species map), the total number of possible events within the species branch is a function of *k*. Since there are *m* species nodes, the total number of events in an event set is bounded by **O**(*h*(*k*)*m*) for some function *h*(*k*). □

#### **Lemma 3**

Given two descriptors *a*=〈*v*,*κ*,*E*〉 and *b*=〈*v*^′^,*κ*^′^,*E*^′^〉 in a Pareto-optimal set, *a*+*b* can be computed in time **O**(*j*(*k*)*m*^2^) for some function *j*(*k*).

#### *Proof*

Adding the event counts and computing the product of the LCT counts takes constant time. The time required to compute the new event set is dominated by the cost of enumerating all pairs of events, one from each of the two original event sets. By Lemma 2, there are **O**(*h*(*k*)*m*) events in each set. Thus, the cost is bounded by *O*(*h*^2^(*k*)*m*^2^), which is **O**(*j*(*k*)*m*^2^) for some function *j*(*k*). □

#### **Lemma 4**

Given two sets $\mathcal {A}$and $\mathcal {B}$ of Pareto-optimal descriptors, the set $\mathcal {A} \otimes \mathcal {B}$can be computed in time **O**(*p*(*k*)*m*^6^) for some function *p*(*k*).

#### *Proof*

Libeskind-Hadas et al. [14] showed that $\mathcal {A} \otimes \mathcal {B}$ can be computed in **O**(*K*^2^ log*K*) time, where *K* is a bound on the size of a Pareto-optimal set. However this algorithm does not keep track of events. While combining two event counts takes constant time, we must now combine two descriptors. If *M* bounds the time to combine two items (in this case descriptors), then this algorithm runs in time **O**(*K*^2^(*M*+ log*K*)). In this case, *K* is **O**(*g*(*k*)*m*^2^) by Lemma 1, and *M*, the cost of combining two descriptors, is **O**(*j*(*k*)*m*^2^) by Lemma 3. Therefore, the total running time is bounded by **O**(*p*(*k*)*m*^6^) for some function *p*(*k*). □

#### **Lemma 5**

Given two sets $\mathcal {A}$and $\mathcal {B}$ of Pareto-optimal descriptors, the set $\mathcal {A} \oplus \mathcal {B}$can be computed in time **O**(*p*(*k*)*m*^6^) for some function *p*(*k*).

#### *Proof*

$\mathcal {A} \oplus \mathcal {B}$ can be computed in time **O**(*K*^2^(*M*+ log*K*)) via a simple modification of the procedure for $\mathcal {A} \otimes \mathcal {B}$ in Libeskind-Hadas et al. [14]. Therefore, as in Lemma 4, the total running time is bounded by **O**(*p*(*k*)*m*^6^) for some function *p*(*k*). □

#### **Theorem 1**

The running time of the DLCparETO algorithm is bounded by **O**(*f*(*k*)*m*^7^) for some function *f*(*k*).

#### *Proof*

We bound the asymptotic running time by considering each of the steps of the algorithm.

**Step 1**: The LCA mapping from the gene tree to the species tree can be computed in time **O**(*mn*) [17]. Since the number of gene nodes mapped to a given species node is bounded by **O**(*k*), there are at most **O**(*km*) gene nodes, and this step takes time **O**(*km*^2^).

**Step 2**: Next, we construct the *C*^*s*^(*t*,*b*) table for each species node. Consider a single species branch which, as noted above, has **O**(*k*) gene tree nodes. Therefore, the time required to generate all possible sub-locus maps, sub-partial orders, and the events that they induce is bounded by a function of *k*. Computing $\mathcal {A}$ for a single tile and *C*^*s*^(*t*,*b*) across all tiles requires repeatedly applying ⊕, each of which takes time **O**(*p*(*k*)) by Lemma 5 (since *m*=1). Therefore, the total time to construct the *C*^*s*^(*t*,*b*) table over all species is bounded by **O**(*q*(*k*)*m*) for some function *q*(*k*).

**Step 3**: The dynamic programming step constructs the *F*^*s*^(*t*,·) table. The number of top loci patterns (and bottom loci patterns) for any species is bounded by some function *r*(*k*), and thus the size of the table is bounded by *r*(*k*)*m*. If *s* is a leaf, then computing *F*^*s*^(*t*,·) takes constant time. Otherwise, computing *F*^*s*^(*t*,·) requires intermediate variables $\phantom {\dot {i}\!}F^{s}(t,b) = F^{s'}(b,\cdot) \otimes F^{s^{\prime \prime }}(b,\cdot) \otimes C^{s}(t,b)$. Each *F*^*s*^(*t*,*b*) can be computed in time **O**(*p*(*k*)*m*^6^) by Lemma 4, and there are up to *r*(*k*) such variables. Similarly, computing *F*^*s*^(*t*,·)=⊕_*b*:(*t*,*b*)∈**RLP**(*s*)_*F*^*s*^(*t*,*b*) requires applying ⊕ over all possible bottom loci. Each ⊕ operation can be computed in time **O**(*p*(*k*)*m*^6^) by Lemma 5, and there are up to *r*(*k*) such operations. Since there are *m* species nodes, the total time to construct the *F*^*s*^(*t*,·) table is bounded by **O**(*t*(*k*)*m*^7^) for some function *t*(*k*).

Therefore, the total running time is bounded by **O**(*km*^2^+*q*(*k*)*m*+*t*(*k*)*m*^7^), which is **O**(*f*(*k*)*m*^7^) for some function *f*(*k*). □

#### **Theorem 2**

Given a Pareto-optimal set of LCTs, the Equivalent Region Partition Problem can be solved in time **O**((*g*^2^(*k*)*m*^4^ log(*g*(*k*)*m*)) for some function *g*(*k*).

#### *Proof*

By Lemma 1, the number of distinct descriptors in a Pareto-optimal set is bounded by **O**(*g*(*k*)*m*^2^). Thus, from the proof of Theorem 3.3 in Libeskind-Hadas et al. [14], it follows that the regions can be computed in time **O**(*g*^2^(*k*)*m*^4^ log(*g*(*k*)*m*)). □

Theorems 1 and 2 together show that the Equivalent Region Partition problem is fixed-parameter tractable. If DLCparETO tracks only event counts rather than full descriptors, as events are not needed for the ERP problem, then its running time is bounded by **O**(*f*(*k*)*m*^5^ log(*km*)) for some (different) function *f*(*k*).

### Computing event support

While the number of distinct MPRs can grow exponentially with *m* and *n* [11], the total number of distinct events is bounded by **O**(*h*(*k*)*m*) for some function *h*(*k*) (Lemma 2). To identify well-supported events, we consider two definitions. Given a descriptor with LCT count *κ*, an event is said to have *region support* *s*,0≤*s*≤1 (with respect to the region) if the event is found in at least a fraction *s* (inclusive) of LCTs. Given an event cost space with *k* regions, an event is said to have *consensus support* *s*,0≤*s*≤1 (with respect to the event cost space) if the event is found in *any* LCT in at least a fraction *s* (inclusive) of the regions. Note that these are only some of many possible measures of event support.

## Results and discussion

### Setup

To demonstrate the utility of our algorithm, we analyzed a biological dataset of 5351 gene families across 16 fungal genomes [16]. Gene trees were reconstructed using RAxML [18] then corrected using TreeFix [19]. We ran DLCparETO using duplication and loss costs ranging from 0.2 to 5 (relative to the unit cost of coalescence), then aggregated results across all gene families. The specific range of event costs can affect the number of regions and the level of event support. Furthermore, for species that are closely related, we might expect that duplications and losses are more costly than coalescences. Therefore, we also investigated a “clamped” cost range, with duplication and loss costs ranging from 1 to 5 (relative to the unit cost of coalescence).

Experiments were performed on a 64-core cluster consisting of four AMD Opteron 6276 CPUs, each with 16 cores at 2.3GHz, and a total of 512GB of DDR3-1600 RAM. The results here exclude 14 (∼0.26*%*) gene families for which DLCparETO used more than the allocated 12 h or 8GB of RAM; such gene families are often very large or highly incongruent to the species tree. The remaining gene families had mean (median, max) leaf sets of 15.2 (16, 84) genes, with a standard deviation (sd) of 7.2 genes. In general, *k*, the maximum number of nodes at the top or bottom of any species branch was small (mean 1.5, sd 0.8, median 1, max 9), so the algorithm ran to completion quickly (mean 1.11 sec, sd 25.95 sec, median 0.09 sec, max 24.68 min).

Despite the different underlying evolutionary models, we compared our results to similar analysis on the DTL model [14], which considered a subset of 3399 gene families from 20 randomly sampled species across the tree of life [20]. The gene families had mean (median, max) leaf sets of 8.9 (6, 73) genes, with a standard deviation of 8.4 genes, and experiments considered transfer and loss costs ranging from 0.5 to 2 (relative to the unit cost of duplication). Previous analyses of multiple optima for a single setting of event costs suggested that the space of MPRs under the two models can be both similar and different [11, 21].

### Number of equivalent regions

Under the DLC model, the majority (68.6*%*; clamped: 87.2*%*) of gene families induce one Pareto-optimal event count and thus one region, suggesting that, for this dataset, a single setting of event costs may be sufficient. However, a small minority (7.3*%*, clamped: 8.1*%*) of families induce at least one region with zero area (e.g. lines or points); such regions would be difficult to discover through an ad-hoc choice of event costs. These results are in stark contrast to the DTL model, in which few (14.2*%*) families induce a single region and most (54.1*%*) families have at least one region with zero area.

**Fig. 6:**
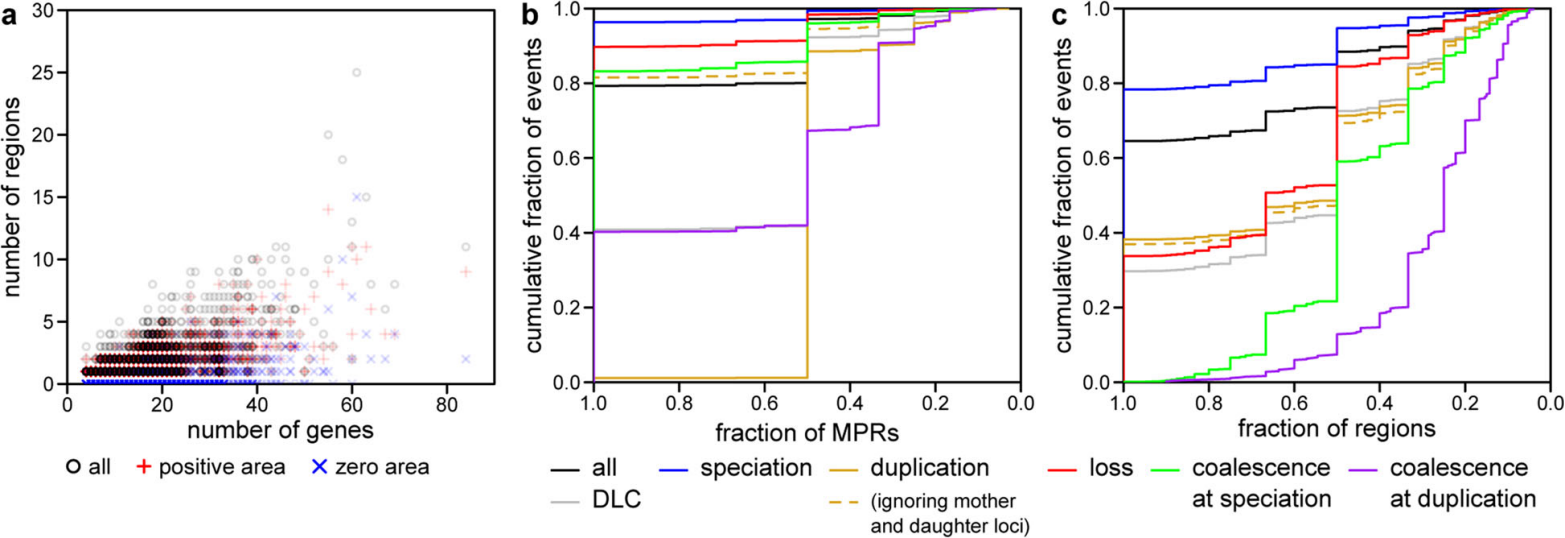
Event support for the real fungal dataset. **a** For all gene families, the number *y* of equivalent regions compared to the number *x* of extant genes (all regions, black circles; positive area regions, red plus sign; zero area regions, blue multiplication sign). **b** Event support as measured by the fraction of MPRs. Coordinate (*x*,*y*) indicates that fraction *y* of events are found in at least fraction *x* of MPRs, with the plot being left-continuous (such that the highest *y* for each *x* should be read). An event is included if it is found in any MPR across the range of event costs, and the same event in multiple regions is treated as separate events. Over all gene families, 142,051 speciations; 33,376 duplications; 18,373 losses; 10,030 coalescence at speciations; and 984 coalescence at duplications are inferred. **c** Event support as measured by the fraction of regions. An event is included if it is found in any MPR across the range of event costs, and the same event in multiple regions is treated as the same event. Over all gene families, 81,965 speciations; 15,929 duplications; 10,554 losses; 5475 coalescence at speciations; and 537 coalescence at duplications are inferred

But, as in the DTL model, the number of regions grows as a function of tree size (Fig. 6a), suggesting that a single cost setting can pose a problem for larger datasets. One possible explanation for this growth is that larger gene trees allow for more incongruence with the species tree and thus more ways to explain this incongruence through different reconciliations.

### Event support within a region

Many events have high region support (Fig. 6b). Including events that are found in any MPR across the range of event costs and treating the same event in multiple regions as separate events, we inferred 204,814 events across all gene families. Of these, 79.3*%* are fully supported (that is, found in all MPRs in its region) and 97.2*%* have at least 50% support.

However, in many applications, we are interested only in the history of speciations, duplications, and losses and consider deep coalescences as nuisance events. For example, orthologous and paralogous pairs of genes are determined from speciations and duplications, respectively. Disaggregated by event type, speciations are the best supported, followed by losses, then coalescence at speciations; at least 83.2*%* of these events are supported regardless of threshold. In contrast, duplications and coalescence at duplications are poorly supported. But a duplication in a species branch can yield two locus maps that differ only in the lineages labeled with the mother locus and daughter locus; these are treated as two distinct duplications in our analysis. If we treat these duplications as the same event, as they result in the same sets of paralogs, then duplication support increases dramatically to levels similar to all other event types except coalescence at duplications. Using clamped costs increased support for speciations, losses, and coalescence at speciations and had little effect on duplications and coalescence at duplications.

These results support those of Du et al. [11], which investigated five settings of event costs and relied on 100 uniformly sampled MPRs per setting. Our analysis depends neither on sampling the event costs nor MPRs and thus presents a fuller picture of event support.

### Event support across regions

Many events also have high consensus support though at lower levels than region support (Fig. 6c). Of the 114,460 events inferred across all gene families, 64.5*%* are fully supported (that is, found in at least one MPR across all regions) and 88.4*%* have at least 50% support.

Disaggregated by event type, across most support thresholds, speciations are the best supported, followed by losses, duplications, coalescence at speciations, and coalescence at duplications. However, unlike for region support, ignoring mother and daughter loci for duplications had little effect. Using clamped costs decreased support for speciations and duplications but increased support for losses and had little effect on coalescences.

Interestingly, events have much higher support under the DLC model than the DTL model. In the latter, only 10.3*%* of events are fully supported and 45.7*%* have at least 50% support. While the DTL model also found speciations to be the most supported type of event, duplications are better supported than losses.

## Conclusions

In this work, we have presented an algorithm for understanding the relationship between event costs and maximum parsimony reconciliations under the DLC model. This algorithm allows users to systematically explore event costs over a range of biologically realistic parameters. If many gene families induce a single Pareto-optimal event count over the range, as in our case study, users can be certain that using a single event cost setting is sufficient when inferring MPRs. Alternatively, gene families that induce several Pareto-optimal event counts may require sampling multiple event cost settings.

In addition, our algorithm allows users to gain insight into event support. In our case study on a biological dataset of 16 fungi, we found that speciations, and thus orthologs, tend to be robust both across MPRs within a single region and across regions within a given range of events costs. While coalescence events had low support under both definitions, these types of events are often of less interest as they do not contribute to the duplication and loss history of a gene family. More research is needed to determine whether these results generalize to other datasets.

While we have focused on MPRs in this work, future development might allow for suboptimal reconciliations, which can be beneficial when the true evolutionary history of a gene family is not parsimonious. For example, To et al. [15] showed how to compute *ε*-Pareto-optimal reconciliations, which allowed for an “over-cost” *ε*.

## Supplementary information

**Additional file 1** Inferring Pareto-Optimal Reconciliations across Multiple Event Costs under the Duplication-Loss-Coalescence Model — Supplementary Material.

## Data Availability

The algorithms are part of the DLCpar software, which is freely available for download at http://www.cs.hmc.edu/~yjw/software/dlcpar.

## Abbreviations

DL: duplication-loss
DLC: duplication-loss-coalescence
DTL: duplication-transfer-loss
ERP: equivalent region partition
LCA: lowest common ancestor
LCT: labeled coalescent tree
MPR: maximimum parsimony reconciliation
MSC: multispecies coalescent
