# Supplementary material for: Inferring Pareto-optimal reconciliations across multiple event costs under the duplication-loss-coalescence model
Source: BMC Bioinformatics. 2019 Dec 17;20(Suppl 20):639. doi: 10.1186/s12859-019-3206-6 (PMC6916210; doi:10.1186/s12859-019-3206-6)
Supplement: Supplementary file 1 — Additional file 1 Inferring Pareto-Optimal Reconciliations across Multiple Event Costs under the Duplication-Loss-Coalescence Model — Supplementary Material. [file 12859_2019_3206_MOESM1_ESM.pdf]

Inferring Pareto-Optimal Reconciliations across Multiple Event Costs under the Duplication-Loss-Coalescence Model – Supplementary Material

Ross Mawhorter, Nuo Liu, Ran Libeskind-Hadas, and Yi-Chieh Wu

This supplement extends the work of Wu et al. (2014) and Du et al. (2019) to track Pareto-optimal event counts and descriptors. Significant new additions and changes are highlighted.

S1 DLC Reconciliation

S1.1 Preliminaries

Throughout this work, the term *tree* refers to a rooted binary tree. Given a tree T, let $V(T)$ denote its node set and $E(T)$ denote its branch set. Let $L(T) \subset V(T)$ denote its leaf set, $I(T) = V(T) \setminus L(T)$ denote its set of internal nodes, and $r(T) \in I(T)$ denote its root node. For node $v \in V(T)$, let $c(v)$ denote its set of children, $p(v)$ denote its parent, and $e(v)$ denote the branch $(p(v), v)$. Define $\leq_T$ ($<_T$) to be the partial order on $V(T)$, where given two nodes u and v of T, $u \leq_T v$ ($u <_T v$) if and only if u is on the unique path between $r(T)$ and v (and $u \neq v$). The partial order $\geq_T$ ($>_T$) is defined analogously. In such a case, u is said to be a (strict) *ancestor* of v and v a (strict) *descendant* of u.

Let a *species tree* S depict the evolutionary history of a set of species, and let a *gene tree* G depict the evolutionary history of a set of genes sampled from these species. To compare a gene tree with a species tree, let a *leaf map* $Le\colon L(G) \to L(S)$ label each leaf of the gene tree with the leaf of the species tree from which the gene was sampled.

S1.2 Species Maps and Speciation Nodes

Definition S1.1 (Species Map). Given G, S, and Le, let a *species map* $\mathcal{M} : V(G) \to V(S)$ map each node of G to the a node of S subject to the following constraints:

1. If $g \in L(G)$, then $\mathcal{M}(g) = Le(g)$.
2. If $g \in I(G)$, then for each $g' \in c(g)$, $\mathcal{M}(g) \leq_S \mathcal{M}(g')$.

Constraint 1 asserts that $\mathcal{M}$ extends the leaf map Le. Constraint 2 asserts that $\mathcal{M}$ satisfies the temporal constraints implied by S.

An internal gene node $g \in I(G)$ is said to be a *speciation node* with respect to species map $\mathcal{M}$ if for each child $g' \in c(g), \mathcal{M}(g') \neq \mathcal{M}(g)$. Given a map $\mathcal{M}$, some nodes may initially be hidden in a gene tree due to losses and deep coalescence. Such "implied speciation nodes" are added to each gene branch that spans multiple branches of the species tree as follows: Locate each non-root internal node $g \in I(G) \setminus \{r(G)\}$ such that either (a) $p(\mathcal{M}(g)) \neq \mathcal{M}(p(g))$ or (b) $p(g)$ is not a speciation node and $\mathcal{M}(g) \neq \mathcal{M}(p(g))$. For each such gene node g, introduce a new node h and replace edge $(p(g), g)$ with the pair of edges $(p(g), h)$ and (h, g), and define $\mathcal{M}(h) = p(\mathcal{M}(g))$. This process is repeated until there exists no node g that satisfies the conditions above. Additionally, if a speciation node has multiple children, then these children must map to different species. Note that if $\mathcal{M}$ is the lowest common ancestor (LCA) map, then this constraint is ensured by construction since the contrapositive is true. That is, if $\mathcal{M}$ is the LCA map, then for a gene node g with children g' and g'', if $\mathcal{M}(g') = \mathcal{M}(g'') = s$, it must be that $\mathcal{M}(g) = s$, and g is not a speciation node. Otherwise, to ensure this constraint, implied speciation nodes are added as follows: Locate each internal node $g \in I(G)$ with children g' and g'' such that g is a speciation node and $\mathcal{M}(g') = \mathcal{M}(g'')$. For each child $g' \in c(g)$, introduce a new node h and replace edge (g, g') with the pair of edges (g, h) and (h, g'),

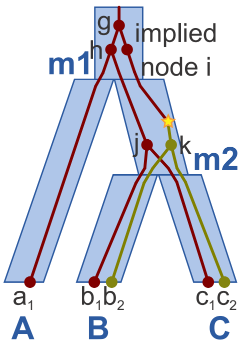

	$\mathcal{M}$	$\mathcal{L}$
g	$m1$	1
h	$m1$	1
i	$m1$	1
j	$m2$	1
k	$m2$	2
a_1	A	1
b_1	B	1
b_2	B	2
c_1	C	1
c_2	C	2

$\mathbb{L} = \{1,2\}$

	$mother\_loci$
$m1$	$\{\}$
$m2$	$\{1\}$
A	$\{\}$
B	$\{\}$
C	$\{\}$

	N	D
$m2$,1	$\{j\}$	$\{k\}$

$k <_{\mathcal{O}} j$

Figure S1. The labeled coalescent tree. Evolution is represented using the LCT, which consists of a species map $\mathcal{M}$, a locus set $\mathbb{L}$, a locus map $\mathcal{L}$, and a partial order $\mathcal{O}$. Sets $mother\_loci(\cdot)$ of loci and $N(\cdot,\cdot)$ and $D(\cdot,\cdot)$ of nodes necessary for the partial order are also shown. [Figure and caption adapted with permission from Du et al. (2019) and Wu et al. (2014).]

and define $\mathcal{M}(h) = \mathcal{M}(g)$. This process is repeated until there exists no node g that satisfies the conditions above. Throughout the remainder of this work, a gene tree G with a species map $\mathcal{M}$ is assumed to contain all implied nodes.

Next, we define some useful sets. Given a species node $s \in V(S)$ and a species map $\mathcal{M}$, let $nodes(s) = \{\, g \mid g \in V(G);\ \mathcal{M}(g) = s\,\}$ denote the set of gene nodes that map to s; $bottoms(s) = \{g \mid g \in nodes(s);\ g \in L(G) \vee \forall g' \in c(g), g' \notin nodes(s)\}$ denote the subset of $nodes(s)$ that are leaves or whose children map to descendants of s; and $tops(s) = bottoms(p(s))$ if $s \neq r(S)$ and $tops(s) = \{r(G)\}$ otherwise. Note that $bottoms(s)$ and $tops(s)$ can be viewed as the set of gene nodes at the "bottom" or "top" of species branch $e(s)$, respectively.

S1.3 The Labeled Coalescent Tree

The *labeled coalescent tree* (LCT, Figure S1) formalizes the notion of a reconciliation in the DLC model.

Definition S1.2 (Labeled Coalescent Tree)**.** Given G, S, and Le, a *labeled coalescent tree (LCT)* for $\langle G, S, Le\rangle$ is a tuple $\langle \mathcal{M}, \mathbb{L}, \mathcal{L}, \mathcal{O}\rangle$, where

- $\mathcal{M}\colon V(G) \to V(S)$ is a **species map** that maps each node of G to a node of S.
- $\mathbb{L} \subset \mathbb{N}$ is a **locus set**, a finite set of natural numbers, each representing a locus that has evolved within the gene family.
- $\mathcal{L}\colon V(G) \to \mathbb{L}$ is a **locus map** that maps each node of G to a locus in $\mathbb{L}$.
- $\mathcal{O}$ is a **partial order** on $V(G)$ that represents the relative times of nodes. For each species node $s \in V(S)$, let $mother\_loci(s) \subset \mathbb{L}$ be the set of loci that yield a new locus in species s:

$$mother\_loci(s) = \{\, \mathcal{L}(g) \mid g \in I(G);\ \exists g' \in c(g), \mathcal{M}(g') = s, \mathcal{L}(g') \neq \mathcal{L}(g)\,\}.$$

Then for each species node $s \in V(S)$ and each locus $l \in mother\_loci(s)$, consider the set of gene nodes $O(s,l) = N(s,l) \cup D(s,l)$, where $N(s,l)$ contains "original" gene nodes that map to species s and locus l, descend from locus l, and have multiple children:

$$N(s,l) = \{\, g \mid g \in V(G) \setminus \{r(G)\};\ \mathcal{M}(g) = s;\ \mathcal{L}(g) = l;\ \mathcal{L}(p(g)) = l;\ |c(g)| > 1\,\},$$

and $D(s,l)$ contains "duplication" gene nodes that map to species s and not locus l but immediately descend from locus l:

$$D(s,l) = \{\, g \mid g \in V(G) \setminus \{r(G)\};\ \mathcal{M}(g) = s;\ \mathcal{L}(g) \neq l;\ \mathcal{L}(p(g)) = l\,\}.$$

Note that the sets $N(s,l)$ and $D(s,l)$ are disjoint. Now consider a total order on $D(s,l)$; this order introduces $|D(s,l)|+1$ bins in which each node in $N(s,l)$ may occur. The total order on $D(s,l)$ and the partition of $N(s,l)$ represent the relative times of duplication nodes as well as the relative times of original nodes with respect to duplication nodes. Define $<_{\mathcal{O}}$ to be the partial order on $O(s,l)$, where given two nodes $g, g' \in O(s,l)$, $g \neq g'$, then $g <_{\mathcal{O}} g'$ if and only if g precedes g' in time. Note that no order is induced on nodes of $N(s,l)$ in the same bin.

The LCT is subject to the following constraints:

1. For each $g, g' \in L(G)$, $g \neq g'$, if $\mathcal{M}(g) = \mathcal{M}(g')$, then $\mathcal{L}(g) \neq \mathcal{L}(g')$.
2. For each $l \in \mathbb{L}$, there exists a $g \in V(G)$ such that $\mathcal{L}(g) = l$.
3. For each $l \in \mathbb{L}$, there exists exactly one $g \in V(G)$ such that $L(g) = l$ and either $g = r(G)$ or $\mathcal{L}(p(g)) \neq l$.
4. For each $s \in V(S)$, each $l \in \mathit{mother\_loci}(s)$, and each $g, g' \in O(s,l)$, $g \neq g'$, if $g <_{\mathcal{O}} g'$, then $g \ngeq_G g'$.

Constraint 1 asserts that extant genes (leaves) mapped to the same extant species (leaves) belong to different loci. Constraint 2 asserts that $\mathbb{L}$ includes only loci used by at least one gene. Constraint 3 asserts that every locus is created only once. Constraint 4 asserts that $\mathcal{O}$ satisfies the temporal constraints implied by G.

Because the locus set $\mathbb{L}$ is defined by the locus map $\mathcal{L}$, we often represent an LCT using the reduced tuple $\langle \mathcal{M}, \mathcal{L}, \mathcal{O} \rangle$.

S1.4 Evolutionary Events in the LCT

For a set of gene nodes $W \subseteq V(G)$, let $\mathit{loci}(W) = \{ \mathcal{L}(g) \mid g \in W \}$ denote the set of associated loci.

Definition S1.3 (Events)**.** Given G, S, Le, an LCT $\langle \mathcal{M}, \mathcal{L}, \mathcal{O} \rangle$ for $\langle G, S, Le \rangle$ induces the following events:

- **Speciation**: For each internal species node $s \in I(S)$, each locus $l \in \mathit{loci}(\mathit{bottoms}(s))$ induces a speciation event.

  This speciation event is represented by a tuple $\langle s, A \rangle$, where A is the set of speciation nodes mapped to s and locus l.

- **Duplication**: For each species node $s \in V(S)$ and locus $l \in \mathit{mother\_loci}(s)$, each gene node $d \in D(s,l)$ induces a duplication event.

  This duplication event is represented by a tuple $\langle s, d, B \rangle$, where B is the set of gene lineages in species s at locus l contemporaneous with the duplication [see (Deep) Coalescence at Duplication]. Note that the contemporary lineages are not needed to include this duplication in a count, only to represent this duplication. Thus, different partial orders can result in different duplications despite the fact that d occurs along the same gene branch.

- **Loss**: For each species node $s \in V(S)$, each locus $l \in \mathit{loci}(\mathit{tops}(s) \cup \mathit{nodes}(s)) \setminus \mathit{loci}(\mathit{bottoms}(s))$ induces a loss event.

  A MPR never loses a locus that that is created via a duplication in the same species. Therefore, in practice, we must only consider each locus $l \in \mathit{loci}(\mathit{tops}(s)) \setminus \mathit{loci}(\mathit{bottoms}(s))$. This loss event is represented by a tuple $\langle s, A \rangle$, where A is the set of speciation nodes mapped to $p(s)$ and locus l.

- **(Deep) Coalescence at Speciation**: For each species node $s \in V(S)$ and each locus $l \in \mathit{loci}(\mathit{tops}(s))$, let $C(s,l)$ denote the set of gene lineages at locus l at the speciation creating s (from $p(s)$). That is, $C(s,l)$ is the subset of gene branches $(g, g') \in E(G)$ such that $g \in \mathit{tops}(s)$, $g' \in \mathit{nodes}(s)$, and $\mathcal{L}(g) = l$. If $|C(s,l)| > 1$, then $C(s,l)$ induces a coalescence at speciation event with $|C(s,l)| - 1$ extra lineages. Each extra lineage incurs the coalescence cost.

  This coalescence at speciation event is represented by a tuple $\langle s, C(s,l) \rangle$.

- **(Deep) Coalescence at Duplication**: For each species node $s \in V(S)$ and each locus $l \in mother\_loci(s)$, let $start(s, l)$ denote the set of "starting" gene lineages in species s at locus l. That is, $start(s, l)$ is the subset of gene branches $(g, g') \in E(G)$ such that $g \in \{r(G)\} \cup tops(s) \cup D(s, l)$, $g' \in nodes(s)$, and $\mathcal{L}(g) = l$. Then, for each duplication node $d \in D(s, l)$, let $K(s, l, d)$ denote the set of gene lineages in species s at locus l contemporaneous with duplication d, where a lineage is contemporaneous if it starts before and ends after the duplication node. This set is determined as follows: Start with $K(s, l, d) = start(s, l)$. Then process nodes $g \in O(s, l)$ in the order specified by $\mathcal{O}$: for each g, remove $e(g)$ from $K(s, l, d)$, and if $g \in N(s, l)$, then for each $g' \in c(g)$, add $e(g')$ to $K(s, l, d)$. Once $g = d$ (duplication reached), if $|K(s, l, d)| > 1$, then $K(s, l, d)$ induces a coalescence at duplication event with $|K(s, l, d)| - 1$ extra lineages. Each extra lineage incurs the coalescence cost.

  This coalescence at duplication event is represented by a tuple $\langle s, K(s, l) \rangle$.

In practice, for a gene node g, rather than use its (possibly arbitrary) name, we report the leaves that are descendants of g.

S1.5 Maximum Parsimony Reconciliations

Let C_D, C_L, and C_C denote the positive real-number costs associated with duplication, loss, and coalescence events, respectively. The traditional goal of DLC reconciliation is to find a most parsimonious reconciliation:

Problem S1.1 (Most Parsimonious Reconciliation (MPR) Problem)**.** Given G, S, Le, C_D, C_L, and C_C, find an LCT for $\langle G, S, Le \rangle$ with minimum reconciliation cost.

Problem S1.2 (Restricted Most Parsimonious Reconciliation (MPR) Problem)**.** Given G, S, Le, C_D, C_L, and C_C, find an LCT for $\langle G, S, Le \rangle$ with minimum reconciliation cost *subject to the condition that the reconciliation between the locus tree and species tree is the LCA map.*

For a comparison of the problems, see Section S2.1 of Wu et al. (2014). In brief, using simulations, we found that the true (simulated) LCT satisfies the additional constraint in almost all gene families. For simplicity, in the main manuscript and henceforth, the term MPR refers to an LCT that solves the restricted MPR problem.

An MPR must satisfy certain properties.

Theorem S1.1 (Optimal Species Maps)**.** *The species map $\mathcal{M}^*$ is optimal if and only if $\mathcal{M}^*$ is the lowest common ancestor (LCA) map.*

Theorem S1.2 (Optimal Locus Maps)**.** *Given a species map $\mathcal{M}$, if the locus map $\mathcal{L}^*$ is optimal, then*

- *Each gene branch $e(g) \in E(G)$ has at most one duplication.*
- *For each species node $s \in V(S)$ and each gene node $g \in nodes(s) \setminus bottoms(s)$ internal to the species branch, if g' and g'' denote the children of g, then at most one of the two children branches $e(g')$ or $e(g'')$ has a duplication.*

Theorem S1.3 (Optimal Partial Orders)**.** *Given a species map $\mathcal{M}$ and locus map $\mathcal{L}$, if the partial order $\mathcal{O}^*$ is optimal, then for each species $s \in V(S)$ and each locus $l \in mother\_loci(s)$, duplications are placed as early in the species branch as possible. That is, for each original node $g \in N(s, l)$ and each duplication node $d \in D(s, l)$, $g <_{\mathcal{O}^*} d$ if and only if $g \leq_G d$.*

See Wu et al. (2014) and Du et al. (2019) for proofs.

S2 The DLCparETO Algorithm

In this section, we need to keep track of the LCT $\langle \mathcal{M}, \mathcal{L}, \mathcal{O} \rangle$ currently under consideration. In particular, many functions are parameterized by $\mathcal{M}$, $\mathcal{L}$, and/or $\mathcal{O}$. Because the optimal species map is the LCA map, the dependency on $\mathcal{M}$ is omitted. However, dependencies on $\mathcal{L}$ or $\mathcal{O}$ are denoted using subscripts.

S2.1 Definitions

In general, maps are denoted in calligraphic font (e.g. $\mathcal{A}$) and sets denoted in bold font (e.g. $\mathbf{A}$).

Given a tree T, define the following:

- **distance**: Given two nodes $u, v \in V(T)$, let the *distance* $d_T(u,v)$ from u to v be the length, in edges, of the unique path from u to v.
- **lowest common ancestor**: Given a non-empty set of nodes $W \subseteq V(T)$, let the *lowest common ancestor* $lca_T(W)$ be the unique shared ancestor of W of maximum distance from $r(T)$.

Given a gene tree G, species tree S, species map $\mathcal{M}$, and species node $s \in V(S)$, define the following:

- **subtrees**: Let *subtrees*(s) denote the subtrees of G within $e(s)$. That is, each subtree $T \in$ *subtrees*(s) is a subtree of G, where the subtree root is a node $r \in \{r(G)\} \cup$ *tops*(s) and the subtree leaves are the nodes $g \in$ *bottoms*(s) that are descendants of r.
- **sub-locus maps**: Let $\mathcal{L}^s\colon$ *tops*$(s) \cup$ *bottoms*$(s) \to \mathbb{L}$ denote a *sub-locus map* that maps each gene node in species branch $e(s)$ to a locus in $\mathbb{L}$. Let $\mathbf{L}(s)$ denote the set of sub-locus maps for s.
- **local orders** and **sub-partial orders**: Given a set of sub-locus maps $\mathbf{L}(s)$:
  - Given a sub-locus map $\mathcal{L}^s \in \mathbf{L}(s)$ and a locus $l \in$ *mother_loci*$_{\mathcal{L}^s}(s)$, let $\mathcal{O}^{s,l}_{\mathcal{L}^s}$ denote a *local order* that partially orders gene nodes $O_{\mathcal{L}^s}(s,l)$ in species branch $e(s)$.
  - Given a sub-locus map $\mathcal{L}^s \in \mathbf{L}(s)$, let $\mathcal{O}^{s}_{\mathcal{L}^s}$ denote a *sub-partial order* that, for each $l \in$ *mother_loci*$_{\mathcal{L}^s}(s)$, partially orders gene nodes $O_{\mathcal{L}^s}(s,l)$ in species branch $e(s)$. Note that combining local orders $\mathcal{O}^{s,l}_{\mathcal{L}^s}$ for all $l \in$ *mother_loci*$_{\mathcal{L}^s}(s)$ yields a sub-partial order $\mathcal{O}^{s}_{\mathcal{L}^s}$.
  - Given a sub-locus map $\mathcal{L}^s \in \mathbf{L}(s)$, let $\mathbf{O}(s,\mathcal{L}^s)$ denote the set of sub-partial orders for s and $\mathcal{L}^s$.
  - Given a sub-locus map $\mathcal{L}^s \in \mathbf{L}(s)$, let $\mathbf{D}(s,\mathcal{L}^s)$ denote the set of Pareto-optimal descriptors for s and $\mathcal{L}^s$ across the set $\mathbf{O}(s,\mathcal{L}^s)$ of sub-partial orders.
- **relative locus pairs**, **top loci**, and **bottom loci**: Given a set of sub-locus maps $\mathbf{L}(s)$:
  - Given a sub-locus map $\mathcal{L}^s \in \mathbf{L}(s)$, let $(\mathcal{TL}^s, \mathcal{BL}^s)$ denote a *relative locus pair* that compactly represents the locus assignments at *tops*(s) and *bottoms*(s) (see main text, Section 3 for details on the compact representation). That is, $\mathcal{TL}^s\colon$ *tops*$(s) \to \mathbb{L}$ denotes a top-locus map (or *top loci*) that maps each gene node at the top of species branch $e(s)$ to a (relative) locus in $\mathbb{L}$, and $\mathcal{BL}^s\colon$ *bottoms*$(s) \to \mathbb{L}$ denotes a bottom-locus map (or *bottom loci*) that maps each gene node at the bottom of species branch $e(s)$ to a (relative) locus. The sub-locus map $\mathcal{L}^s$ is said to be an *underlying sub-locus map* for $(\mathcal{TL}^s, \mathcal{BL}^s)$, and $\mathcal{L}^s$ is said to *induce* $(\mathcal{TL}^s, \mathcal{BL}^s)$.
  - Let $\mathbf{TL}(s)$ denote the set of top loci, $\mathbf{BL}(s)$ denote the set of bottom loci, and $\mathbf{RLP}(s)$ denote the set of relative locus pairs for s. That is, these are the sets of $\mathcal{TL}^s$, $\mathcal{BL}^s$, and $(\mathcal{TL}^s, \mathcal{BL}^s)$ for all $\mathcal{L}^s \in \mathbf{L}(s)$.
- **Pareto-optimal descriptors**: Let $\mathbb{P}$ denote the set of all Pareto-optimal descriptors. That is, the set $v(\mathbb{P})$ of event counts contains all vectors $v = \langle d, \ell, c\rangle$ such that $d, \ell, c \in \mathbb{N}$ and every event count v is Pareto-optimal with respect to $v(\mathbb{P})$.
  - Given a set of relative locus pairs $\mathbf{RLP}(s)$, let $C^s\colon \mathbf{RLP}(s) \to \mathbf{P} \subset \mathbb{P}$ map each relative locus pair to a set of Pareto-optimal descriptors. Specifically, for a relative locus pair $(\mathcal{TL}^s, \mathcal{BL}^s) \in \mathbf{RLP}(s)$, consider the set of LCTs with species map $\mathcal{M}$, a sub-locus map $\mathcal{L}^s \in \mathbf{L}(s)$ that induces $(\mathcal{TL}^s, \mathcal{BL}^s)$, and a sub-partial order $\mathcal{O}^{s}_{\mathcal{L}^s} \in \mathbf{O}(s,\mathcal{L}^s)$. $C^s(\mathcal{TL}^s, \mathcal{BL}^s)$ denotes the set of Pareto-optimal descriptors for this set of LCTs.
  - Given a set of relative locus pairs $\mathbf{RLP}(s)$, let $F^s\colon \mathbf{RLP}(s) \to \mathbf{P} \subset \mathbb{P}$ map each relative locus pair to a set of Pareto-optimal descriptors. Specifically, for a relative locus pair $(\mathcal{TL}^s, \mathcal{BL}^s) \in \mathbf{RLP}(s)$, $F^s(\mathcal{TL}^s, \mathcal{BL}^s)$ denotes the set of Pareto-optimal descriptors for the subtree of S rooted at s with relative locus pair $(\mathcal{TL}^s, \mathcal{BL}^s)$ for s. Furthermore, for top loci $\mathcal{TL}^s \in \mathbf{TL}(s)$, $F^s(\mathcal{TL}^s, \cdot)$ denotes the set of Pareto-optimal descriptors for the subtree of S rooted at s with top loci $\mathcal{TL}^s$ for s.

S2.2 Pseudocode

The main algorithm is shown in Algorithm S1, with helper algorithms in Algorithms S2–S3. For simplicity, details are omitted for cases when a species branch contains no gene tree nodes.

Algorithm S1 Main Algorithm

---

function DLCparETO(G, S, Le)
input gene tree G, species tree S, leaf mapping Le
output set of Pareto-optimal descriptors for G and S

▷ set optimal species map to LCA map (see Theorem S1.1), and decompose gene tree (Figure 2A)
for each gene node $g \in V(G)$ in post-order **do**
  if $g \in L(G)$ **then**
    Set $\mathcal{M}^*(g) = Le(g)$
  else
    Set $\mathcal{M}^*(g) = lca_S(\{\mathcal{M}^*(g'), \mathcal{M}^*(g'')\})$, where g' and g'' denote the children of g
Prune species tree S to the subtree rooted at $\mathcal{M}^*(r(G))$ ▷ gene tree evolves within subtree of species tree
Add implied speciation nodes to gene tree G using $\mathcal{M}^*$ (Supplemental Section S1.2)
Decompose gene tree G into disjoint subtrees $subtrees(s)$

▷ construct tiles for each species (Figure 2B,D)
for each species node $s \in V(S)$ in pre-order **do**
  ▷ get set of top loci
  if $s = r(S)$ **then**
    Assign $r(G)$ to an arbitrary locus
    Set $\mathbf{TL}(s)$ to the set (of size 1) that includes this locus assignment
  else
    Set $\mathbf{TL}(s) = \mathbf{BL}(p(s))$

  ▷ construct all tiles starting from set of top loci, find set of Pareto-optimal descriptors for tiles
  Set $\mathbf{L}(s) =$ GetAllLocusMaps(G, S, $\mathcal{M}^*$, s, $subtrees(s)$, $\mathbf{TL}(s)$) ▷ find set of sub-locus maps
  for each sub-locus map $\mathcal{L}^s \in \mathbf{L}(s)$ **do**
    Initialize $\mathbf{D}(s, \mathcal{L}^s) = \varnothing$ ▷ set of Pareto-optimal descriptors
    Set $\mathbf{O}(s, \mathcal{L}^s) =$ GetAllPartialOrders(G, $\mathcal{M}^*$, s, $\mathcal{L}^s$) ▷ find set of sub-partial orders
    for each sub-partial order $\mathcal{O}^s_{\mathcal{L}^s} \in \mathbf{O}(s, \mathcal{L}^s)$ **do**
      Find set E of events and event count $v = \langle d, \ell, c \rangle$ for $\langle \mathcal{M}^*, \mathcal{L}^s, \mathcal{O}^s \rangle$
      Set $a = \langle v, 1, \{(e, 1) \mid e \in E\} \rangle$ ▷ construct descriptor
      Update $\mathbf{D}(s, \mathcal{L}^s) = \mathbf{D}(s, \mathcal{L}^s) \otimes \{a\}$

  ▷ compact tiles, find set of Pareto-optimal descriptors for compact tiles (Figure 2C,D)
  Initialize $\mathbf{BL}(s) = \varnothing$, $\mathbf{RLP}(s) = \varnothing$
  Initialize C^s to an empty map
  for each sub-locus map $\mathcal{L}^s \in \mathbf{L}(s)$ **do**
    Find relative locus pair $(\mathcal{TL}^s, \mathcal{BL}^s)$ for $\mathcal{L}^s$
    Add $\mathcal{BL}^s$ to $\mathbf{BL}(s)$, and $(\mathcal{TL}^s, \mathcal{BL}^s)$ to $\mathbf{RLP}(s)$
    if $C^s(\mathcal{TL}^s, \mathcal{BL}^s)$ is empty **then**
      Initialize $C^s(\mathcal{TL}^s, \mathcal{BL}^s) = \varnothing$
    Update $C^s(\mathcal{TL}^s, \mathcal{BL}^s) = C^s(\mathcal{TL}^s, \mathcal{BL}^s) \otimes \mathbf{D}(s, \mathcal{L}^s)$

▷ merge Pareto-optimal descriptors across species (Figure 2E)
Initialize F^s to an empty map
for each species node $s \in V(S)$ in post-order **do**
  for each top loci $\mathcal{TL}^s \in \mathbf{TL}(s)$ **do**
    if $s \in L(S)$ **then**
      Set $\mathcal{BL}^s$ to single element of $\mathbf{BL}(s)$
      Set $F^s(\mathcal{TL}^s, \cdot) = C^s(\mathcal{TL}^s, \mathcal{BL}^s)$
    else
      Set s' and s'' to be the children of s
      for each bottom loci $\mathcal{BL}^s \in \mathbf{BL}(s)$ **do**
        Set $F^s(\mathcal{TL}^s, \mathcal{BL}^s) = F^{s'}(\mathcal{BL}^s, \cdot) \otimes F^{s''}(\mathcal{BL}^s, \cdot) \otimes C^s(\mathcal{TL}^s, \mathcal{BL}^s)$
      Set $F^s(\mathcal{TL}^s, \cdot) = \oplus_{\mathcal{BL}^s:(\mathcal{TL}^s, \mathcal{BL}^s) \in \mathbf{RLP}(s)} F^s(\mathcal{TL}^s, \mathcal{BL}^s)$

Set $s = r(S)$
Set $\mathcal{TL}^s$ to single element of $\mathbf{TL}(s)$
return $F^s(\mathcal{TL}^s, \cdot)$

Algorithm S2 Enumerate Sub-Locus Maps

function GETALLLOCUSMAPS(G, S, $\mathcal{M}$, s, $subtrees(s)$, $\mathbf{TL}(s)$)
input gene tree G, species tree S, species map $\mathcal{M}$, species node s
  set $subtrees(s)$ of subtrees for species s,
  set $\mathbf{TL}(s)$ of top loci for species s
output set of sub-locus maps $\mathbf{L}(s)$ for species s

Find DP ▷ find all combinations of duplication placements over branches of $subtrees(s)$ (see Theorem S1.2)

Initialize $\mathbf{L}(s) = \varnothing$
for each sub-locus map $\mathcal{TL}^s \in \mathbf{TL}(s)$ **do** ▷ combine set of top loci and set of duplication placements
  for each combination of duplication placements $dp \in DP$ **do**
    Initialize an empty sub-locus map $\mathcal{L}^s$
    for each subtree $T \in subtrees(s)$ **do**
      for each gene node $g \in V(T)$ in pre-order **do** ▷ "evolve" locus down subtree
        if $g \in \{r(G)\} \cup tops(s)$ **then** ▷ set locus assignments for root nodes of subtrees
          Set $\mathcal{L}^s(g) = \mathcal{TL}^s(g)$
        else ▷ set locus assignments for non-root nodes of subtrees
          if dp has a duplication on $e(g)$ **then**
            Set $\mathcal{L}^s(g)$ to a new locus ▷ a new locus is required by LCT Constraint 5
          else
            Set $\mathcal{L}^s(g) = \mathcal{L}^s(p(g))$ ▷ locus is equal to parent locus
    if $s \notin L(S) \vee (s \in L(S) \wedge \forall g, g' \in bottoms(s)$ s.t. $g \neq g', \mathcal{L}^s(g) \neq \mathcal{L}^s(g'))$ **then** ▷ LCT Constraint 3
      Add $\mathcal{L}^s$ to $\mathbf{L}(s)$
return $\mathbf{L}(s)$

Algorithm S3 Enumerate Sub-Partial Orders for a Sub-Locus Map

function GETALLPARTIALORDERS(G, $\mathcal{M}$, s, $\mathcal{L}^s$)
input gene tree G, species map $\mathcal{M}$, species node s
sub-locus map $\mathcal{L}^s$ for species s
output set $\mathbf{O}(s, \mathcal{L}^s)$ of sub-partial orders for species s and sub-locus map $\mathcal{L}^s$

for each locus $l \in mother\_loci_{\mathcal{L}^s}(s)$ **do** ▷ find local orders for this mother locus
  Initialize an empty set $\mathbb{O}^{s,l}_{\mathcal{L}^s}$ of local orders
  for each local order $\mathcal{O}^{s,l}_{\mathcal{L}^s} \in$ ORDERHELPER($start_{\mathcal{L}^s}(s,l), D_{\mathcal{L}^s}(s,l), O_{\mathcal{L}^s}(s,l)$, empty ordered set) **do**
    Add $\mathcal{O}^{s,l}_{\mathcal{L}^s}$ to $\mathbb{O}^{s,l}_{\mathcal{L}^s}$

▷ combine local orders across mother loci
Set $\mathbf{O}(s, \mathcal{L}^s)$ by combining $\mathbb{O}^{s,l}_{\mathcal{L}^s}$ for all $l \in mother\_loci_{\mathcal{L}^s}(s)$
return $\mathbf{O}(s, \mathcal{L}^s)$

iterator ORDERHELPER(G, SL, DN, ON, $\hat{O}$)
input gene tree G, starting lineages SL, duplication nodes DN, original nodes ON, current local order $\hat{O}$
yield updated local order O

Set $PDN = \hat{O} \cap DN$ ▷ placed duplication nodes
Set $PON = \hat{O} \cap ON$ ▷ placed original nodes

if $DN = PDN$ **then** ▷ all duplication nodes have been placed
  Add set $U = ON \setminus PON$ to $\hat{O}$ ▷ add unplaced original nodes
  yield $\hat{O}$
else
  ▷ find unplaced duplication nodes with minimum ancestor constraints (see Theorem S1.3)
  Set $DN' = \arg\min_{g \in DN \setminus PDN} d_G(r, g)$, where $r \in V(G)$ such that $e(r) \in SL$ and $r \leq_G g$
  for each gene node $g \in DN'$ **do**
    Create copy O' of $\hat{O}$
    Find set U of gene nodes $g' \in ON \setminus PON$ s.t. $g' \leq_G g$ ▷ unplaced ancestors of duplication node
    Add U to O' ▷ add partition of original nodes
    Add g to O' ▷ add duplication node
    for each $O \in$ ORDERHELPER(SL, DN, ON, O') **do** ▷ recur to add remaining nodes
      yield O
end iterator

References

Du H, Ong Y. S, Knittel M, Mawhorter R, Liu N, Gross G, Tojo R, Libeskind-Hadas R and Wu Y.-C. 2019. Multiple optimal reconciliations under the duplication-loss-coalescence model. In 17th Asia Pacific Bioinformatics Conference (APBC 2019). Wuhan, China.

Wu Y.-C, Rasmussen M. D, Bansal M. S and Kellis M. 2014. Most parsimonious reconciliation in the presence of gene duplication, loss, and deep coalescence using labeled coalescent trees. Genome Research **24**:475–486.
